# Extracellular Interactions between Fibulins and Transforming Growth Factor (TGF)-β in Physiological and Pathological Conditions

**DOI:** 10.3390/ijms19092787

**Published:** 2018-09-17

**Authors:** Takeshi Tsuda

**Affiliations:** 1 Nemours Cardiac Center, Nemours/Alfred I. duPont Hospital for Children, 1600 Rockland Road, Wilmington, DE 19803, USA; ttsuda@nemours.org; Tel.: +1-(302)651-6677; Fax: +1-(302)651-6601; 2 Department of Pediatrics, Sidney Kimmel Medical College at Thomas Jefferson University, 1025 Walnut Street, Philadelphia, PA 19107, USA

**Keywords:** matricellular protein, extracellular matrix (ECM), epithelial–mesenchymal transition (EMT), remodeling, positive feedback loop, signal transduction

## Abstract

Transforming growth factor (TGF)-β is a multifunctional peptide growth factor that has a vital role in the regulation of cell growth, differentiation, inflammation, and repair in a variety of tissues, and its dysregulation mediates a number of pathological conditions including fibrotic disorders, chronic inflammation, cardiovascular diseases, and cancer progression. Regulation of TGF-β signaling is multifold, but one critical site of regulation is via interaction with certain extracellular matrix (ECM) microenvironments, as TGF-β is primarily secreted as a biologically inactive form sequestrated into ECM. Several ECM proteins are known to modulate TGF-β signaling via cell–matrix interactions, including thrombospondins, SPARC (Secreted Protein Acidic and Rich in Cystein), tenascins, osteopontin, periostin, and fibulins. Fibulin family members consist of eight ECM glycoproteins characterized by a tandem array of calcium-binding epidermal growth factor-like modules and a common C-terminal domain. Fibulins not only participate in structural integrity of basement membrane and elastic fibers, but also serve as mediators for cellular processes and tissue remodeling as they are highly upregulated during embryonic development and certain disease processes, especially at the sites of epithelial–mesenchymal transition (EMT). Emerging studies have indicated a close relationship between fibulins and TGF-β signaling, but each fibulin plays a different role in a context-dependent manner. In this review, regulatory interactions between fibulins and TGF-β signaling are discussed. Understanding biological roles of fibulins in TGF-β regulation may introduce new insights into the pathogenesis of some human diseases.

## 1. Introduction

Transforming growth factor-β (TGF-β) is a multifunctional peptide growth factor that plays a vital role in regulating cell proliferation, differentiation, inflammation, angiogenesis, and tissue repair [1,2]. Dysregulation of TGF-β is known to mediate multiple pathological conditions including tissue fibrosis, chronic inflammation, cardiovascular diseases, and cancer progression [3,4,5,6]. TGF-β activation is regulated at multiple levels and consists of a very complex network [7,8,9]. After mRNA translation, TGF-β precursor is processed and secreted into the extracellular matrix (ECM), where it stays as a latent form with TGF-β binding proteins (LTBP) and latency-associated protein (LAP) [10]. TGF-β has to be cleaved from this latent complex to become a biologically active form, and several proteases including plasmin, matrix metalloproteinase (MMP)-2, and MMP-9 have been identified as latent TGF-β activators [7,11,12]. Free TGF-β executes its role via specific cell surface receptors, TGF-β receptors type I and type II, to phosphorylate Smad proteins as an essential component. TGF-β also activates other signaling cascades, including extracellular-signal-regulated kinase (ERK), c-Jun-N-terminal kinase (JNK), TGF-β-activated kinase 1 (TAK-1), and p38 mitogen-activated protein kinase (MAPK) pathways [13,14]. TGF-β is also known to activate phosphatidylinositol-3 kinase (PI3K)/AKT, Rho-GTPase, Wnt, and Notch pathways, comprising complex signaling cross-talks [15]. Other newly introduced regulatory mechanisms include microRNAs [16], DNA methylation, and histone modification [17]. 

Extracellular regulation of TGF-β ligand activation is mediated, in part, by certain ECM proteins via matrix–matrix interactions [18]. These proteins are called matricellular proteins, which do not contribute to the structural integrity of tissues but mainly play a functional role in activating tissue enzymes and proteases during tissue remodeling [19,20]. Thrombospondins, SPARC (Secreted Protein Acidic and Rich in Cystein), tenascins, osteopontin, and periostin are known to modulate TGF-β activity [18,21]. These ECM proteins not only regulate TGF-β activation but also in turn may be regulated by TGF-β, providing complex cross-talks via matrix–matrix and cell–matrix interactions. Other structural ECM proteins including fibrillin [22,23], fibronectin [24], and decorin [25,26] play a pivotal role in TGF-β activation. A fibulin family is a group of ECM glycoproteins that have both a structural contribution to ECM integrity and a functional role of regulating cell behavior via multiple interactions with other ECM molecules and cell receptors [27,28,29]. Fibulins also assume certain regulatory roles in TGF-β signaling in various ways with or without participating in structural integrity of the tissues. In this review, biological roles of fibulins will be discussed in conjunction with extracellular regulation of TGF-β in pathogenesis of certain human diseases.

## 2. Fibulin Family

**Figure 1:**
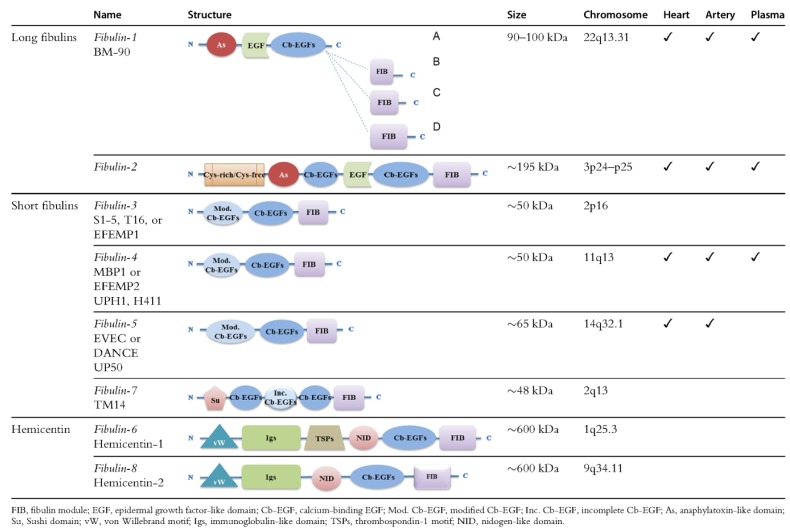
Structure, chromosome localization, and expression of the fibulins [32]. *From Cangemi et al. “Fibulins and their role in cardiovascular biology and disease”, Adv Clin Chem 2014, 67; 245–265*.

Fibulins are a family of eight ECM glycoproteins characterized by a tandem array of calcium-binding epidermal growth factor (cbEGF)-like modules and a homologous C terminal domain (fibulin-type carboxyl-terminus, FC) of 120 to 140 amino acids [29]. The signature structural features (tandem cbEGF and FC) are also present in fibrillins, which constitute the core of microfibrils [30]. The fibulins are found in a variety of tissues in association with diverse supramolecular structures, including elastic fibers, basement membrane networks, fibronectin microfibrils, and proteoglycan aggregates [28,31]. Based upon length and domain structures, these eight fibulins are divided into two subgroups: long fibulins (fibulin-1, -2, -6, and -8) and short fibulins (fibulin-3, -4, -5, and -7) (Figure 1) [31,32,33]. The biological significance of fibulins has been studied through the expression and tissue deposition patterns, in vitro cell culture experiments, the phenotypic assessment of genetically modified experimental animals, and human diseases of specific fibulin mutations.

### 2.1. Long Fibulins

Fibulin-1 and fibulin-2 are the original fibulins larger in size than fibulin-3, -4, and -5 [31]. Recently, fibulin-6 and -8 (hemicentin-1 and -2, respectively) have been added to this group based upon the large molecular mass. 

#### 2.1.1. Fibulin-1

Fibulin-1 (BM-90) is the first fibulin discovered by Argraves and his colleagues as an ECM protein of approximately 90 kDa in size expressed in the ECM of multiple organs [34]. Fibulin-1 is widely expressed in association with basement membrane [35] and matrix fibers, especially elastic fibers through amorphous elastin components [36]. Fibulin-1 has been shown to interact with integrin, proteoglycan aggrecan and versican [37], nidogen [38], and e ADAMTS (A Disintegrin And MMP with ThromboSpondin motifs)-1 [39], suggesting its role in tissue remodeling. Fibulin-1 is highly upregulated in early embryonic development and is a constituent of most basement membranes [40]. Subsequently, extensive extracellular accumulation of fibulin-1 is shown at the sites of epithelial–mesenchymal transition (EMT), such as endocardial cushion tissue during valvuloseptal formation [40,41]. Fibulin-1 is widely expressed in the connective tissues throughout the body of mouse embryos including lung, intestine, kidney, brain, blood vessels, and liver [42]. In adult human tissue, fibulin-1 is predominantly expressed in connective tissue rich in elastic fibers, such as blood vessels, lungs, and skin [36]. Fibulin-1 is also present in a soluble form in plasma and interacts with fibrinogen [43].

Fibulin-1 null mice display a severe perinatally lethal phenotype involving multiple organ systems consistent with its early onset and severe defects in the basement membranes of many organs including kidneys and lungs resulting in fetal hemorrhage and organ malformations [44]. Another model of fibulin-1 deficient mice by gene trap insertion technique demonstrated that fibulin-1 is essential in cell motility of migrating mesenchymal cells including endocardial cushion cells and neural crest cells during embryonic heart development [45]. With this fibulin-1 deficient mouse model, it was shown that fibulin-1 is required for ADAMTS1-mediated versican cleavage that suppresses trabecular myocyte proliferation during the development of ventricular myocardium, a critical step in forming a myocardial compact zone [46]. It was previously shown in the mouse embryonic kidney that fibulin-1 mediates ADAMTS-1-induced proteoglycan proteolysis through their direct molecular interaction, suggesting a regulatory role of fibulin-1 in the morphogenesis of kidney epithelium [39]. 

Fibulin-1 is known to possess both tumor suppressive and enhancing effects [47]. The expression of fibulin-1 is low in many tumor-derived cell lines, and exogenous fibulin-1 (fibulin-1D) suppresses cell growth and invasion in human fibrosarcoma, suggestive of its inhibitory role for tumorigenesis [48]. Fibulin-1 inhibits in vitro cell adhesion and motility in a cell- and matrix-specific manner; fibronectin is required for fibulin-1 to suppress cell motility of breast cancer [49,50] and ovarian cancer cells [50]. On the other hand, breast cancers exhibit elevated fibulin-1 expression compared with surrounding normal tissue, implicating fibulin-1 as a promoter of breast cancer development and progression [51]. Moll et al. demonstrated that fibulin-1C is preferentially upregulated in ovarian cancer cells, suggesting fibulin-1C, not fibulin-1D, promotes ovarian cancer progression [52]. Thus, cell specificity, ECM microenvironment, and specific domain of the protein all contribute to the complex outcome of fibulin-1 involvement in tumorigenesis [53]. 

#### 2.1.2. Fibulin-2

Fibulin-2 (FBLN-2) was identified from sequence analysis of cDNA clones obtained from a mouse fibroblast library [54]. Fibulin-2 is a dimer of two disulfide-bonded 195-kDa monomers [55]. Fibulin-2 binds to tropoelastin and thus serves as an interface between the elastin core and fibrillin microfibrils during vascular development [54,56,57]. Fibulin-2 also binds to other ECM molecules, including fibronectin, fibrillin-1, fibulin-1, nidogen, laminin, and versican [29]. Fibulin-2 expression partially overlaps with that of fibulin-1 but shows a more restricted tissue distribution pattern [58]. During embryonic development, fibulin-2 shows an abrupt increase in expression in the endocardial cushion tissue in E10.5 mouse embryo [59,60]. Fibulin-2 continues robust expression throughout the development of cardiac valves and septa, coronary vessels, and aortic arch vessels, suggesting its involvement in EMT [60]. Fibulin-2 is also expressed in the developing cartilages and the thin capsule-like connective tissue sheaths covering internal visceral organs including lung, liver, and kidney [42,58]. In adult mouse tissues, fibulin-2 continues to show restrictive expression in cardiac valves, epicardium, endothelial basement membrane of the blood vessels, interstitial tissue of skeletal muscle, cornea, and skin [54]. Fibulin-2 is upregulated in skin wound healing, suggesting its role in tissue remodeling [61]. Fibulin-2 serves as a specific marker of rat liver myofibroblasts distinct from other fibrogenic liver cells (e.g., hepatic satellite cells) [62] and is upregulated in chronic liver fibrosis induced by carbon tetrachloride (CCl_4_) [63]. 

Fibulin-2 null mice show normal phenotype with normal growth, development, and fertility [64], but the phenotypic features may be subtle or transient. Compensatory upregulation of fibulin-1 is seen in aorta and skin tissues [64] and mammary gland [65] of fibulin-2 null mice, which may be responsible for the lack of obvious phenotype. There is a transient and partial abnormality in skin basement membrane formation in the newborn fibulin-2 null mice similar to integrin α3β1 null mice, which show diminished fibulin-2 induction, suggesting fibulin-2 is necessary in supporting integrin α3β1-induced neonatal skin basement membrane stability [66]. 

A biological role of fibulin-2 has been investigated in several human cancer disorders, as fibulin-2 is highly upregulated during EMT. However, the role of fibulin-2 in cancer development is not straightforward; it may inhibit or promote tumorigenesis. In human nasopharyngeal carcinoma, fibulin-2 is indicated to assume tumor-suppressive effects by inhibiting cell growth and proliferation, cell migration and invasion, and angiogenesis by downregulating pro-angiogenesis factors [67]. In Kaposi’s sarcoma, an angioproliferative tumor of vascular endothelial cells, significant downregulation of fibulin-2 is demonstrated in combination with downregulated fibulin-3 and fibulin-5 and upregulation of fibulin-1, suggesting that loss of fibulin-2 compromises the structural integrity of vascular basement membrane due to loss of interactions between fibulin-2 and other ECM proteins, inducing uncontrolled cell proliferation, migration, and invasion [68]. In breast cancer cell lines, fibulin-2 inhibits cancer progression by suppressing cell migration and invasion, demonstrated by the fact that fibulin-2 is significantly reduced in invasive breast cancer cell lines and the reintroduction of fibulin-2 into these cell lines reduced cancer cell motility and invasion [69]. In contrast, fibulin-2 is shown to enhance malignant progression of metastatic lung adenocarcinoma by promoting cross-linking of secreted collagen molecules and tumor cell adherence [70]. Fibulin-2 plays a complex role in cancer development depending upon the cell types, cancer stages, and the degree of malignancy. The recent study by Fontanil et al. showed the interaction between fibulin-2 and ADAMTS-12, a secreted metalloproteinase, promotes antitumor effects in breast cancer cells, while ADAMTS-12 may elicit protumor effects in the absence of fibulin-2 [71]. These interactions may underlie the molecular mechanisms by which some protumor metalloproteinases exert their antitumor activities. 

#### 2.1.3. Fibulin-6 and Fibulin-8

Hemicentins are an ECM glycoprotein in *C. elegans* newly identified by Vogel et al. as an evolutionarily conserved ECM protein with roles in tissue organization, migration, basement membrane invasion, and cell–cell and cell–matrix contacts mainly in epithelial tissues [72]. Because of their molecular structure with typical fibulin modules (cbEGF repeats and FC module), hemicentin-1 and -2 are classified as fibulin-6 and -8, respectively [73,74]. With a molecular size of more than 600 kDa, fibulin-6 and -8 are by far the largest members of the fibulin family. Mutation in fibulin-6 leads to massive blistering in the developing fins of zebrafish, suggesting its role in mesenchymal cell migration and epidermal–dermal junction formation [75]. On the other hand, loss of fibulin-8, only with concomitant loss of fibulin-1, induces the similar blistering phenotype in zebrafish [76]. Further studies are warranted to characterize the biological significance of these newer fibulins.

### 2.2. Short Fibulins

Short fibulins contain no more than six cbEGF domains and consist of fibulin-3 (EFEMP1), fibulin-4 (EFEMP2), fibulin-5 (DANCE or EVEC), and fibulin-7 (TM17) [33]. These short fibulins are newer family members than fibulin-1 and fibulin-2 and have been shown to play multiple roles in elastic tissue formation and tissue remodeling.

#### 2.2.1. Fibulin-3

Fibulin-3 (EFEMP1) was first found to be overexpressed in senescent human fibroblasts established from a Werner syndrome patient with premature aging [77]. Fibulin-3 mutation causes an autosomal dominant macular degenerative disease (Doyne honeycomb retinal dystrophy) [78,79]. Its spatial expression in mouse tissues is primarily within the elastic tissues and basement membranes and is more or less overlapped with that of fibulin-1 and fibulin-4 [30,58]. In the mouse embryo, fibulin-3 is found in developing cartilage and bone [80], suggesting its role in regulating the shaping of the skeletal elements in the body. Fibulin-3 is abundantly expressed in eye and lung and moderately expressed in brain, heart, and kidney in adult mice [78]. 

Fibulin-3 knockout mice show reduced reproductivity; an early onset of aging-associated phenotypes including reduced lifespan, decreased body mass, and reduced hair growth; and spine deformity and decreased bone density but no evidence of macular degeneration [81]. These findings suggest that loss of fibulin-3 function is not the primary mechanism of macular degeneration but that fibulin-3 plays an important role in maintaining the integrity of connective tissues and regulating aging [81]. 

The involvement of fibulin-3 in cancer development is complex and sometimes contradictory as it exhibits both pro- and anti-neoplastic effects depending upon the cell types and developmental stages. In lung adenocarcinoma cells, fibulin-3 demonstrates inhibitory effects on EMT and self-renewal capacity by suppressing β-catenin through insulin-like growth factor-1 receptor (IGF1R)/phosphatidylinositide 3-kinase (PI3K)/AKT signaling pathways [82]. High fibulin-3 levels inhibit progression of breast cancer by suppressing TGF-β-induced EMT, migration, invasion, and endothelial permeability [83]. On the contrary, fibulin-3 is upregulated in advanced pancreatic adenocarcinoma and plays a role in enhancing cancer progression by promoting vascular endothelial growth factor (VEGF)-mediated angiogenesis and inhibiting apoptotic mechanisms [84]. In malignant glioma, increased expression of fibulin-3 is shown to promote tumor progression and invasion by enhancing cell adhesion and migration via Notch signaling [85,86]. Fibulin-3 levels in the blood and pleural fluid are significantly elevated in patients with mesothelioma compared with those with exposure to asbestos and healthy controls, suggesting its clinical value as a biomarker in determining diagnosis and prognosis [87,88].

#### 2.2.2. Fibulin-4

Fibulin-4 (EFEMP2) was characterized as an ECM glycoprotein of ~60 kDa with structural domains similar to fibulin-3 [30]. Fibulin-4 is strongly expressed in the heart; moderately in the skeletal muscle; and weakly in brain, placenta, lung, and pancreas [30,89]. Fibulin-4 is expressed intensely in the outer medial layers toward the adventitia in large blood vessels [90]. Fibulin-4 is also expressed in articular chondrocytes and cultured chondrocyte cells [91]. In primary osteoblast cell culture, proper fibulin-4 fibril formation in the ECM requires the presence of EMILIN (Elastin-Microfibril-Interface-Located-proteIN)-1, a pro-TGF-β processing protein, for modulating collagen homeostasis [92]. Mutations of fibulin-4 cause an autosomal recessive form of cutis laxa syndrome with aortic aneurysms, arterial tortuosity and stenosis, and minor skin involvement, a different phenotype seen in other forms of cutis laxa either by elastin or fibulin-5 mutation in which skin involvement is the most prominent clinical manifestation [93,94]. 

The functional role of fibulin-4 was studied in fibulin-4 deficient mice that exhibit perinatal lethality in association with hemorrhage due to rupture of tortuous and aneurysmal aortic vessels and emphysematous lung [95]. These severely abnormal vascular and lung defects are attributed to defects in elastic fiber formation including coacervation, cross-linking and deposition, and organization processes allowing equal distribution onto microfibrils [33]. Mice homologous for the fibulin-4 reduced expression allele (Fibulin-4^R/R^) survive the perinatal period but show dilatation of ascending aorta and a tortuous and stiffened aorta resulting not only from disorganized elastic fiber assembly but also likely from the dysregulation of the TGF-β signaling pathway [96]. The number of smooth muscle cells in the aortic media is also decreased in fibulin-4^R/R^ mice [97]. During aortic development, fibulin-4 contributes, not only to the formation of elastic fibers, but also to terminal differentiation and maturation of smooth muscle cells in the aortic wall, especially actin cytoskeleton organization [90]. It was shown that fibulin-4 is essential for elastic fiber assembly in the ascending aorta or large conduit arteries but not in the medium sized muscular arteries, suggesting elastin assembly has different requirements depending on vessel types [98]. However, emphysematous lung is not seen in fibulin-4^R/R^ mice. The degree of aortic aneurysm was shown to be inversely proportional to the amount of fibulin-4 available in the tissue, and the reduced fibulin-4 allowed MMP activation, particularly MMP-9, via enhanced TGF-β signaling [99]. Sasaki et al. demonstrated that different mutations in the fibulin-4 gene result in different molecular defects affecting secretion rates, protein stability, cross-linking, and molecules of the TGF-β pathway [100].

The role of fibulin-4 in tumorigenesis has not been fully elucidated. Fibulin-4 mRNA expression is found to be significantly increased in colon tumors [89]. Increased fibulin-4 expression is associated with poor prognosis of human osteosarcoma, and fibulin-4 promotes osteosarcoma cell invasion and metastasis by inducing EMT via the PI3K/AKT pathway [101] and Wnt/β-catenin pathway [102]. However, in human endometrial carcinoma, the fibulin-4 protein expression level is inversely correlated with the malignant phenotype, and fibulin-4 demonstrates inhibitory effects in endometrial carcinoma proliferation, invasion, metastasis, and EMT through the Wnt/β-catenin pathway [103]. 

#### 2.2.3. Fibulin-5

Fibulin-5 (also known as EVEC or DANCE) is 66-kDa in size and was isolated by subtraction hybridization to identify the genes that regulate the transition from quiescent vascular smooth muscle cells to the proliferative state; it was originally identified as a secreted molecule involved in cardiovascular development and remodeling [104,105]. Fibulin-5 is mainly found in elastic fiber-enriched tissues including aorta, lung, uterus, and skin [104,105], and its mutations are associated with an autosomal recessive form of a rare congenital skin anomaly called cutis laxa [106,107] and age-related macular degeneration development [108]. Fibulin-5 is a matricellular protein contributing to the formation of elastogenic tissues and mediating various cellular functions required for tissue development and homeostasis [109]. 

Fibulin-5 is shown as an essential determinant of elastic fiber organization, as fibulin-5 null mice exhibit a severely disorganized elastic fiber system throughout the body including tortuous aorta with loss of compliance, severe lung emphysema, and loose skin. Fibulin-5 may provide anchorage of elastic fibers to cells, thereby acting to stabilize and organize elastic fibers in the skin, lung, and vasculature [110,111]. Fibulin-5 has multiple binding sites with other ECM proteins including fibrillin-1 [112], lysyl oxidase-like protein-1 (LOXL-1) [113], extracellular superoxide dismutase [114], latent TGF-β binding protein (LTBP)-2 [115], and LTBP-4 [116]. These unique binding sites of fibulin-5 suggest its biological role not only in formation of microfibrillar scaffold, deposition of tropoelastin, and assembly of elastic fibers but also in regulation of functional properties including cell–matrix interaction and signal transduction [117,118]. Exaggerated injury-induced vascular remodeling in fibulin-5 null mice is attributed to both loss of structural integrity of the vessel wall and inability to assemble mature elastic fibers within the neointima and loss of direct inhibitory effects of fibulin-5 on smooth muscle cell proliferation and migration [119]. Fibulin-5 inhibits angiogenesis and endothelial cell activities by antagonizing vascular endothelial growth factor (VEGF) signaling independent of its integrin-binding RGD motif, suggesting its role in regulation of angiogenesis [120]. 

As with other fibulins, fibulin-5 also both suppresses and promotes tumorigenesis in a context-specific manner [53,118]. Fibulin-5 mRNA expression is downregulated in the majority of human tumors, particularly in metastatic malignancies of the kidney, breast, ovary, and colon, suggesting its inhibitory role in advanced cancer development [118]. A recent study showed that fibulin-5 is downregulated in human endometrial cancer and that fibulin-5 knockdown in endometrial epithelial cancer cells enhances adhesion and proliferation of cancer cells, indicating its antitumorigenic role in women [121]. On the contrary, fibulin-5 was shown to enhance the malignancy of human fibrosarcoma cells [118] and mammary epithelial cells via promoting EMT [122]. In pancreatic ductal adenocarcinoma, fibulin-5 promotes tumor progression by blocking reactive oxygen species production through competing with fibronectin for integrin binding sites, resulting in increased angiogenesis and tumor growth [123]. The effects of fibulin-5 on cancer development are complex and warrant further investigations.

#### 2.2.4. Fibulin-7

Fibulin-7 (or TM14) is a newly introduced fibulin family for its molecular structure containing cbEGF-like repeats in the center flanked by homologous C-terminal domain and a unique Sushi domain at the N-terminus [124]. Fibulin-7 is a cell adhesion molecule that interacts with other ECM molecules in developing teeth, suggesting its role in differentiation and maintenance of odontoblasts and in dentin formation. Fibulin-7 is also expressed in cartilage, hair follicles, and placenta [124]. In a novel deletion of human chromosome 2q13 associated with craniofacial malformation and congenital heart disease, fibulin-7 was identified as one of the responsible genes for the phenotype as fibulin-7 knockdown in the zebrafish model leads to cardiac and craniofacial defects as well as reduced cartilage deposition in the pharyngeal arches and impaired branchial arch development [125]. Fibulin-7 also plays a role as an angiogenesis inhibitor by promoting endothelial cell adhesion and inhibiting endothelial tube formation via β1-integrin and heparan sulfate receptors [126]. A recent study by Sarangi et al. demonstrated that fibulin-7 and its C-terminal fragment negatively regulate monocyte and macrophage migration, differentiation, and cytokine production, suggesting their potential immunomodulatory role in treating inflammatory diseases [127]. 

## 3. Interaction between Fibulins and TGF-β

Just like many ECM proteins, the expression of fibulins may be regulated by TGF-β during embryonic development, tissue repair, and pathological processes. At the same time, some fibulins modulate TGF-β activation and its downstream signaling in multiple different ways. Below, bidirectional interactions between fibulins and TGF-β signaling with subsequent phenotypical alterations in cell behavior and ECM components are reviewed.

### 3.1. Fibulin-1

Biological interactions between fibulin-1 and TGF-β in tissue remodeling have been studied in respiratory diseases including pulmonary fibrosis and chronic obstructive pulmonary disease (COPD) [128,129,130] and bone metastasis of prostate cancer [131]. TGF-β treatment downregulates fibulin-1 mRNA in airway smooth muscle cells and induces sequestration of soluble fibulin-1 into ECM [128]. In fibulin-1 deficient mice, cigarette smoking, an experimental model for COPD, fails to induce airway inflammation, remodeling, and TGF-β secretion, suggesting some stimulatory role of fibulin-1 over TGF-β release, but the underlying molecular mechanism remains unknown [130]. TGF-β downstream pathway modulated by fibulin-1 was not investigated in this study. TGF-β treatment suppresses fibulin-1 mRNA expression and protein abundance, and fibulin-1 downregulation by TGF-β reduces the ability of human bone marrow stromal cells to induce prostate cancer cell death [131].

### 3.2. Fibulin-2

Bidirectional interaction between fibulin-2 and TGF-β has been studied in fibulin-2 deficient mice. In the experimental myocardial infarction in the mouse model, absence of fibulin-2 prevents the development of progressive ventricular dysfunction and shows a significantly improved survival rate by attenuating upregulation of other ECM protein synthesis commonly required in wound healing process, MMP-2 activation, and TGF-β signaling, suggesting a regulatory role of fibulin-2 in ECM protein synthesis during scar formation after myocardial necrosis [132]. In the angiotensin II (Ang II) infusion model, absence of fibulin-2 inhibits Ang II-induced myocardial hypertrophy and fibrosis in vivo with suppression of TGF-β signaling, indicating a critical role of fibulin-2 in Ang II-induced TGF-β activation and its downstream signaling [133,134]. In isolated mouse cardiac fibroblasts, TGF-β treatment induces upregulation of fibulin-2 and enhanced TGF-β signaling, both of which are totally abolished in fibulin-2 null cells [133]. R-Smad (Smad2) is a principal downstream pathway in Ang II-infusion model when mediated by fibulin-2, but the involvement of ERK1/2, p38MAPK, and TAK1 is variable depending upon the concentration of infused Ang II [133,134]. These studies suggest a presence of TGF-β-induced positive feedback loop or autoregulation mediated by fibulin-2. Fibulin-2 may be enhancing the release of TGF-β from large latent complex in ECM by competing for the TGF-β binding site of fibrillin-1 with other ECM proteins, such as latent TGF-β binding protein (LTBP)-1 [135]. Enhanced TGF-β activation and downstream signaling are noted in combination with fibulin-2 upregulation in advanced human heart failure myocardium [136,137]. Collectively, it is plausible that ECM environment altered by upregulated fibulin-2 contributes, in part, to the pathogenesis of human heart failure via enhancing myocardial TGF-β activation via positive feedback loop. The same principle of autoregulation is also noted in neuronal tissues, where fibulin-2 mediates TGF-β-induced proneurogenic effects in the rat model in vivo and in vitro [138]. 

### 3.3. Fibulin-3

Interaction between fibulin-3 and TGF-β has not been fully understood. Tian et al. demonstrated that fibulin-3 has a potent inhibitory effect on TGF-β signaling in breast cancer development where fibulin-3 interacts with type I TGF-β receptor by blocking receptor complex formation [83]. However, TGF-β-induced fibulin-3 regulation in the ECM microenvironment was not studied. 

### 3.4. Fibulin-4

The regulatory role of fibulin-4 in TGF-β signaling has been studied in human cutis laxa [93,139] and in the mouse models with deficient or reduced fibulin-4 causing upregulation of TGF-β and defect in elastic tissue formation, resulting in aortic aneurysm and arterial tortuosity [95,96]. Increased TGF-β signaling, via upregulation of both TGF-β1 and TGF-β2, is demonstrated in isolated aortic smooth muscle cells in fibulin-4 deficient mice in a dose-dependent manner, indicating an inhibitory effect of fibulin-4 on TGF-β signaling via R-Smad pathway [140]. Enhanced activation of Smad2 is noted in the fibroblast cell extracts from the patients with cutis laxa with fibulin-4 mutation [93]. In contrast, aortic aneurysm in the mouse of smooth muscle cell-specific deletion of fibulin-4 reveals predominantly enhanced ERK1/2 signaling [90]. As fibulin-4 binds to LTBP-1 with high affinity, fibulin-4 may be additionally involved in sequestration of the large latent complex (LLC) into fibrillin microfibrils though LTBP-1 binding [141]. Reduced fibulin-4 expression induces abnormally enhanced TGF-β signaling responsible for the aortic aneurysm, similar to the phenotype seen in Marfan syndrome and fibrillin-1 deficient mice [142,143,144]. Thus, fibulin-4 is a negative regulator of TGF-β signaling, which is completely opposite to fibulin-2. It is not known, however, whether TGF-β can directly regulate fibulin-4 expression. 

### 3.5. Fibulin-5

TGF-β is known to stimulate fibulin-5 transcription and mRNA expression in human lung fibroblasts via PI3K/AKT pathway [145]. TGF-β stimulates murine 3T3-L1 fibroblasts and endothelial cells to synthesize fibulin-5 transcript and protein through a Smad3-independent pathway, indicating fibulin-5 as a TGF-β-inducible target gene that regulates cell growth and motility in a context-specific manner [118,120]. Overexpression of fibulin-5 enhances basal and TGF-β-stimulated activation of ERK1/2 and p38MAPK in 3T3-L1 fibroblasts [118]. Fibulin-5 expression is enhanced by TGF-β in human endometrial epithelial cancer cells [121]. In pancreatic ductal adenocarcinoma, fibulin-5 is produced by stromal cells, and its expression is induced by TGF-β via PI3K/AKT signaling pathway [146], serving as a protumorigenic factor. Fibulin-5 initiates EMT and enhances TGF-β-induced EMT in mammary epithelial cells via an MMP-dependent mechanism, suggesting a positive regulatory role of fibulin-5 in TGF-β signaling [122].

### 3.6. Other Newer Fibulins (Fibulin-6, -7, and -8)

The regulatory role of fibulin-6 in ventricular remodeling after experimental myocardial infarction and its role in cardiac fibroblast migration was investigated [147]. Fibulin-6 is upregulated in the ischemic myocardium, especially in the infarct border zone, but, paradoxically, TGF-β treatment in isolated mouse cardiac fibroblasts inhibits fibulin-6 expression, indicating complex involvement of fibulin-6 in TGF-β signaling [147]. Further investigation by the same group demonstrated that fibulin-6 plays an important role in regulating TGF-β-mediated responses by enhancing TGF-β receptor dimerization and activation to further trigger downstream pathways [148]. The interaction between other newer fibulins, fibulin-7 and -8, and TGF-β has not been identified. 

## 4. Biological Significance of Fibulins and TGF-β Signaling

Fibulins not only participate in ECM formation by organizing structural integrity of basement membrane and elastic fiber tissues. They also regulate a wide spectrum of cellular functions including embryonic development, tissue homeostasis and remodeling after injury, angiogenesis, and tumorigenesis [27,29], the processes also frequently mediated by TGF-β signaling [1,2]. Although complex, fibulins and TGF-β signaling interact in variable ways in certain contexts. The bidirectional interactions between each individual fibulin and TGF-β signaling are summarized in Table 1.

TGF-β suppresses fibulin-1 mRNA expression and protein release in respiratory cells, whereas fibulin-1 has a stimulatory effect on TGF-β release and subsequent airway remodeling [128,129,130,131]. This relationship includes a negative feedback loop between fibulin-1 and TGF-β, although this may be a partial interaction in the diverse network of cross-talks involving fibulin-1 or TGF-β. In contrast, fibulin-2 and TGF-β stimulate in both ways to create a positive feedback loop in mouse cardiac fibroblasts [133] and neuronal cells [138]. TGF-β-mediated positive feedback loop is considered as one pathogenesis of cancer progression [149,150]. For example, cancer progression is promoted by TGF-β-mediated positive feedback loop in colorectal cancer involving miR-1269 [151] and c-KIT signaling in advanced primary hepatocellular carcinoma [152]. Fibulin-2 may play a similar role in certain pathological conditions. Fibulin-4 plays a totally opposite role in TGF-β signaling to fibulin-2 as absence of fibulin-4 induces uncontrolled upregulation of TGF-β [93,95,96,139], but it is unknown whether TGF-β alters fibulin-4 expression. Fibulin-5 expression is enhanced by TGF-β [121,122,142,146], and fibulin-5 promotes TGF-β-induced EMT through activating MMP-2 and -9 in mammary epithelial cells [122], suggesting bidirectional interaction similar to fibulin-2. Fibulin-6 plays a role in negative feedback loop in regulating TGF-β-mediated profibrotic response in neonatal mouse ventricular cardiac fibroblasts [148], similar to fibulin-1. Each fibulin appears to have a unique biological profile in relation to TGF-β signaling in a context-dependent manner.

## 5. Conclusions

Fibulins play a dual role as a structural ECG protein and a matricellular protein. Fibulins bind to multiple ECM molecules to participate in organizing microenvironments affecting tissue integrity and regulating cell behaviors in both physiological and pathological conditions in a context-dependent manner. TGF-β regulates expression of fibulins in variable ways, whereas secreted fibulins modulate TGF-β signaling. In particular, fibulin-2 and -5 promote TGF-β-mediated positive feedback loop in certain pathological conditions. Fibulins may become relevant therapeutic targets in certain human disease processes including cancer, chronic fibrotic disorders, and heart failure. Cross-talks between fibulins and TGF-β have been identified in certain disease pathogeneses, but the level of our understanding is still at the developing stage. Further research endeavors are encouraged to delineate the underlying mechanisms of these molecular cross-talks. 

## Figures and Tables

**Table 1:** Bidirectional Interactions between Fibulins and TGF-β.

	Fibulin → TGF-β Signaling	TGF-β → Fibulin Expression
Fibulin-1	Enhances TGF-β in airway smooth muscle cells [130]	Inhibits fibuin-1 in airway smooth muscle cells [128]; bone marrow stromal cells [131]
Fibulin-2	Enhances TGF-β in mouse cardiac fibroblasts [133]; mouse myocardium in vivo [133,134]; adult rat neuronal stem cells [138]	Enhances fibulin-2; mouse cardiac fibroblasts [133]; adult rat neuronal stem cells [138]
Fibulin-3	Inhibits TGF-β in breast cancer cells [83]	Unknown
Fibulin-4	Inhibits TGF-β in mouse aorta [96,140]; human cutis laxa [93,139]	Unknown
Fibulin-5	Enhances TGF-β signaling in mammary epithelial cells in MMP-dependent manner [122]	Enhances fibulin-5 expression in human lung fibroblasts [142]; human endometrial epithelial cancer cells [121]; mammary epithelial cells [122]; pancreatic ductal carcinoma cells [146]
Fibulin-6	Enhances TGF-β signaling in cardiac fibroblasts [148]	Inhibits fibulin-6 expression in mouse cardiac fibroblasts [147]
